# The Efficiency of Anterior Repositioning Splints in the Management of Pain Related to Temporomandibular Joint Disc Displacement with Reduction

**DOI:** 10.1155/2018/9089286

**Published:** 2018-02-21

**Authors:** Malgorzata Pihut, Malgorzata Gorecka, Piotr Ceranowicz, Mieszko Wieckiewicz

**Affiliations:** ^1^Department of Prosthodontics, Consulting Room of Temporomandibular Joint Dysfunction, Jagiellonian University Medical College, 4 Montelupich St., 31-155 Krakow, Poland; ^2^University Dental Clinic, Jagiellonian University Medical College, 4 Montelupich St., 31-155 Krakow, Poland; ^3^Department of Physiology, Jagiellonian University Medical College, 16 Grzegorzecka St., 31-531 Krakow, Poland; ^4^Department of Experimental Dentistry, Faculty of Dentistry, Wroclaw Medical University, 26 Krakowska St., 50-425 Wroclaw, Poland

## Abstract

**Background and Objective:**

Intra-articular temporomandibular disorders are often related to pain in the area of the temporomandibular joint, ear, and temple. The aim of the study was to investigate the efficiency of anterior repositioning splints in decreasing pain related to temporomandibular joint disc displacement with reduction.

**Methods:**

The research material consisted of 112 patients, aged 24 to 45 years, of both genders, who reported for treatment at the Consulting Room of Temporomandibular Joint Dysfunctions at the Jagiellonian University in Cracow between 2014 and 2016 due to pain in the area of the temporomandibular joint(s) and noise(s) of temporomandibular joint(s) present during jaw movements with comorbid contracture of masticatory muscles. Subjects were examined according to the Diagnostic Criteria for Temporomandibular Disorders (DC/TMD) protocol and, after diagnosis of painful disc displacement with reduction and masticatory muscle contracture, they were assigned randomly to either the study or control groups (56 patients in each). In the study group, we used an anterior repositioning splint on the full lower arch for about 20 hours usage over a 4-month period. In the control group, a noninvasive therapy was applied using a biostimulation laser over 12 sessions performed every second day on the area of both temporomandibular joints with mouth open and while performing muscle self-exercises with a dominant protrusive position of the mandible. Pain intensity was evaluated using the Verbal Numerical Rating Scale (VNRS) immediately before the treatment and then after 4 and 16 weeks. The obtained data were analyzed using the Mann–Whitney *U* test (*p* ≤ 0.005).

**Results:**

The VNRS values reported during the final examination for the study group were significantly lower than for the control group (*p*=0.0004).

**Conclusions:**

The anterior repositioning splint is an efficient tool in decreasing pain related to disc displacement with reduction. This trial is registered with Clinicaltrials.gov NCT03057262.

## 1. Introduction

Intra-articular temporomandibular disorders present as various pathological conditions, often concerning derangements of the condyle-disc complex [1, 2]. They occur because the physiological relationship between the articular disc and the condyle head changes. According to the Diagnostic Criteria for Temporomandibular Disorders (DC/TMD), we can distinguish four basic types of articular disc displacements: disc displacement with reduction, disc displacement with reduction with intermittent locking, disc displacement without reduction with limited opening, and disc displacement without reduction without limited opening [3].

Many studies confirm that disc displacement with reduction (DDwR) is the most prevalent derangement of the condyle-disc complex [4–6]. The DC/TMD defines DDwR as an intracapsular biomechanical disorder when, in closed mouth position, the disc is in an anterior position relative to the condylar head, and the disc reduces upon opening of the mouth (medial and lateral displacement of the disc may also be present; clicking, popping, or snapping noises may occur with disc reduction) [3]. DDwR could be the result of masticatory muscle contracture. Acoustic symptoms such as clicking, popping, and snapping are evidence of disc displacement with reduction. Clicking occurs when the condylar head of the mandible skips the rear edge of the displaced articular disc during mouth opening and/or closing. Afterwards, the disc does not return to the correct position in relation to the condyle when the mandible is again, upon closure, in the central position. Clicking may occur in the initial, middle, and final phase of the mandible opening movement. Different studies have reported that pain is a comorbid symptom of temporomandibular joint (TMJ) disc displacement [7, 8]. When the disc is anteriorly displaced, the ligaments of the rear disc are elongated, stretched, and damaged, and the bilaminar zone is compressed; and thus, pain can be generated. Sometimes acoustic symptoms are accompanied by pain located in the area of the temporomandibular joints and surrounding tissues, intensifying when the patient opens their mouth and chews food. Over the years, many studies have confirmed the efficacy of anterior repositioning splints (ARS) for the management of TMJ discs displacements, but the number of studies simultaneously evaluating the level of pain is limited [9, 10].

Therefore, the aim of the study was to investigate the efficiency of anterior repositioning splints in the alleviation of pain related to temporomandibular joint disc displacement with reduction.

## 2. Materials and Methods

This is a prospective outcome study which consisted of 112 subjects of both genders with unilateral or bilateral disc displacement(s) with reduction and pain in the area of TMJ. Patients were recruited from the Consulting Room of Temporomandibular Joint Dysfunction at the Jagiellonian University in Cracow during the years 2014–2016 and were included in the study if they met the following criteria: (1) unilateral or bilateral presence of clicking, popping, and/or snapping noise(s) detected with palpation during opening or closing or lateral or protrusive movements in TMJ(s); (2) in the previous 30 days, any TMJ noise(s) present with jaw movement or function; (3) unilateral or bilateral pain in the area of TMJ(s); (4) presence of masticatory muscle contracture during palpation; (5) full dentition or single tooth loss; (6) good general health; (7) positive mandible protrusion test; (8) no contraindications for laser therapy; and (9) patient consent to be involved in the study. Other patients representing TMJ intra-articular disorders who had undergone treatment during the years 2014–2016 were excluded because of partial tooth loss or edentulism, contraindications for laser therapy as well as absence of appropriate symptoms and/or consent to be involved in the study.

Clinical assessment of temporomandibular joints and masticatory muscles was performed by one experienced, self-trained examiner according to DC/TMD recommendations [3]. TMJ noise(s) had persisted for between 3 and 18 months prior to the beginning of the study among included subjects. All patients reported pain in the area of TMJ(s) and positive mandible protrusion test, resulting in the disappearing of the clicking, popping, and/or snapping during mouth opening or closing or lateral movements from protrusive incisal positioning of the mandible. This test was a clue to use the anterior repositioning splint. Patients were randomly assigned to the study group or control group (56 patients in each). Both groups were informed about prevention for disc(s) displacement with reduction. In the study group, we used a typical acrylic anterior repositioning splint fabricated in tete-a-tete (incisal) jaw position, covering the full lower teeth arch to recapture a displaced disc and decrease the level of pain. ARS was recommended for 20-hour use for four months. In the control group, we used a biostimulation laser (Terapus 2, Accuro, Poland), wavelength 808 nm, power 32 J, over 12 sessions (the duration of each session was 3 min 45 s), performed every other day, on the area of both temporomandibular joints (distance to the skin was 1 cm) with opened mouth and systematic performance of TMJ and masticatory muscle self-exercises twice a day for 5 minutes for 16 weeks, according to the TMJ self-mobilization described by Shaffer et al. [11].

Pain level was evaluated using the Verbal Numerical Rating Scale (VNRS). VNRS comprises assessment that is based on an 11-point numerical scale (0–10) in combination with a color-coded scale, in which an increase in the score is accompanied by an increase in color intensity indicated on the scale. Clinical examinations of TMJ noise(s) and pain level evaluations were performed immediately before the treatment (examination I) and then during follow-up visits after 4 weeks (examination II) and 16 weeks (examination III). At the beginning of the treatment, each ARS was adjusted, and upon follow-up visits, occlusion was checked using clinical observations and marking paper to prevent occlusal changes.

The study protocol was conducted in accordance with the Declaration of Helsinki and approved by the Local Bioethical Committee (no. 122.6120.43.2016). All patients provided written informed consent prior to participating in the study.

The obtained data were compared using the Mann–Whitney *U* test because of noncompliance with the normal distribution in the tested groups (*p* ≤ 0.005).

## 3. Results

**Figure 1:**
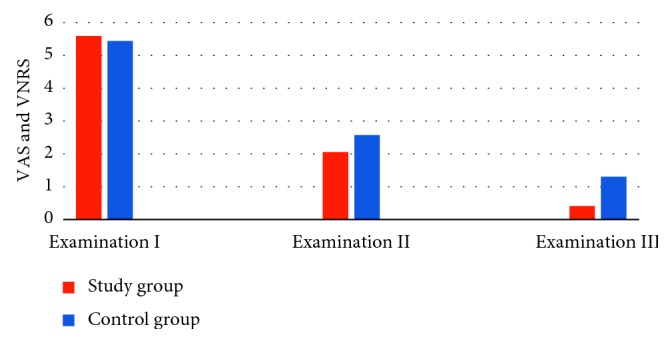
The values of VNRS reported in examinations I, II, and III.

A total of 112 subjects were included in the study: 83 women and 29 men, aged 24–45 years (mean age: 31). The study group (SG) included 40 women and 16 men, and the control group (CG) contained 43 women and 13 men. The clinical findings collected during follow-up visits in SG and CG and their specification are presented in Tables 1 and 2. SG participants of both genders most often complained of unilateral acute pain in the area of TMJ which corresponded to other clinical findings, for example, pain occurring during chewing or jaw movements, pain during palpation, impaired mandible movement, referral of pain within the head, and unilateral clicking in TMJ. The frequency of the above symptoms in SG decreased with the progress of the applied intervention. CG female participants most often complained of unilateral mild pain in the area of TMJ, and CG male participants most often complained of unilateral acute pain in the area of TMJ, which in both genders corresponded to other clinical findings, for example, pain occurring during chewing or jaw movements, pain during palpation, impaired mandible movement, referral of pain within the head, and unilateral clicking in TMJ. The frequency of the above symptoms in CG decreased with the progress of the applied interventions. There were no occlusal changes among participants during ARS therapy. Pain levels reported by patients during follow-up visits are presented in Figure 1. The mean value of VNRS obtained during examination I in SG was 5,589 points and in CG was 5,436. These results did not differ significantly (*p*=0.5015), which indicates that groups are similar in terms of reported pain levels. The VNRS values collected during examination II are lower across the two groups in comparison to examination I (SG was 2,054, and CG was 2,571), and they differ significantly (*p*=0.00048). The values collected during examination III are much lower across the two groups in comparison to examination I; namely, the mean value of reported VNRS for SG was 0.411, and for CG was 1.303. These mean values differ significantly (*p*=0.0004). The statistical analysis is presented in Table 3, and it showed a significant decrease in reported VNRS values during follow-up visits. These analyzes indicate that anterior repositioning splints were an effective tool in pain relief in the group of patients with disc(s) displacement with reduction.

## 4. Discussion

Disc displacement with reduction is the most common derangements of the condyle-disc complex [12]. There are several forms of these derangements, but anterior and anterior-medial displacements are the most frequent [12–14]. Disc displacements may be partial or complete, depending on their extension. Trauma, anatomy of TMJ, bruxism, stress, masticatory muscle contracture, and abnormal dental occlusion may lead to elongation of the disc ligaments and indirectly to disc displacements and excessive load within temporomandibular joints and retrodiscal tissues [7, 15–19].

A serious limitation of this study was the use of only clinical findings without magnetic resonance imaging (MRI) for ruling in a diagnosis of DDwR, because the sensitivity of this method without imaging is 0.34 [3]. According to DC/TMD, imaging is the reference standard for this diagnosis to increase validity, but in Poland, it is not a standard procedure to refer a patient for MRI without serious medical indications; otherwise, he/she will be forced to cover the fee for any such imaging, because the national healthcare system will not cover these costs (the Polish private health insurance sector is underdeveloped). Therefore, we have to base diagnoses on clinical findings.

The use of ARS in the treatment of disc displacement plays an important role, due to TMJ tissue unloading including retrodiscal tissues and insertions of selected masticatory muscles to articular discs [10, 19, 20]. The occlusal splint therapy which was carried out by the authors with the use of ARS was dependent on the results of a positive mandible protrusion test, resulting in the disappearance of the noise(s) during mouth opening from the protrusive incisal position of the mandible. There is some dispute among researchers and clinicians concerning the efficiency of treatment with the use of repositioning splints, with some researchers reporting that occlusal splints are not useful in the treatment of all derangements of the condyle-disc complex. Badel et al. performed a study concerning the effectiveness of Michigan-type splints in displaced disc recapture [21]. The study showed that this splint is not an efficient tool in disc recapture in cases with disc displacement with or without reduction. Conti et al. proved that a positive result from a clinical procedure using an occlusal splint is related to the splint design [22]. Therefore, the practitioner has to correctly choose the design of the occlusal splint upon clinical manifestation of the temporomandibular disorders.

On the other hand, randomized clinical trials performed by Schiffman et al. and Haketa et al. showed that occlusal splint therapy could be an efficient method in displaced disc recapture and also pain level alleviation [23, 24]. In both clinical trials, the authors compared occlusal splint therapy with other treatment options, including conservative and surgical approaches, and concluded that all of these are efficient in the management of intra-articular TMJ disorders.

The assumption behind the control group design was to use noninvasive intervention with low risk of side effects to reduce TMJ(s) pain. Therefore, laser biostimulation combined with TMJ and masticatory muscle self-exercises was a suitable solution. The setup of 12 laser sessions, performed every other day on the area of both temporomandibular joints, is a standard and valid procedure in the Consulting Room of Temporomandibular Joint Dysfunction at the Jagiellonian University for TMJ(s) pain alleviation. Furthermore, regular performance of exercises by the patient provides long-term biomechanical stability to impaired TMJ(s) and masticatory muscle function. The combination of noninvasive techniques could be a sufficient solution to reduce pain among patients with temporomandibular disorders [20].

Invasive treatment such as arthroscopies, arthrocentesis, and other surgical techniques are still considered after failure of conservative management [20, 25]. Invasive procedures are always associated with risk, such as lesion of the articular structures and facial nerve or perforation of the mandibular fossa. However, a few authors suggest the implementation of arthrocentesis to both joints as a good solution for healing retrodiscal tissues [26, 27]. Pain relief in this group of patients is one of the main goals of treatment and the most common cause of patients reporting to the doctor. Evaluation of the efficacy of ARS for the treatment of DDwR showed a significant reduction of pain in the area of the temporomandibular joints. Mean VNRS values were much lower in SG treated with anterior repositioning splints than in CG. Therefore, we can assume that this treatment option is effective upon painful disc displacement with reduction. Management of pain related to TMJ disc displacement using ARS has been also positively evaluated by other authors [28–30].

## 5. Conclusion

Considering the limitation of the study, we can state that the anterior repositioning splint is an efficient tool in decreasing pain related to disc displacement with reduction.

## Figures and Tables

**Table 1:** The results of clinical findings collected during follow-up visits within the study group.

Parameters of pain occurring in the area of TMJ			Women			Men		
			Examination			Examination		
			I	II	III	I	II	III
Pain	Unilateral	Acute	24	6	1	8	4	1
		Mild	6	2	0	5	2	1
	Bilateral	Acute	2	0	2	1	0	0
		Mild	8	5	0	2	1	0
Pain that occurs during food chewing or jaw movements			28	11	1	14	10	1
Pain during palpation			20	16	1	13	16	0
Impaired movement of the mandible			38	3	1	24	11	3
Referral of pain within the head			29	12	0	15	7	2
Clicking in temporomandibular joint		Unilateral	28	24	4	11	5	0
		Bilateral	12	8	2	5	2	1

**Table 2:** The results of clinical findings collected during follow-up visits within the control group.

Parameters of pain occurring in the area of TMJ			Women			Men		
			Examination			Examination		
			I	II	III	I	II	III
Pain	Unilateral	Acute	12	6	1	7	5	3
		Mild	26	12	6	3	3	2
	Bilateral	Acute	3	1	1	1	0	0
		Mild	2	1	1	2	1	0
Pain that occurs during food chewing or jaw movements			26	19	5	11	10	1
Pain during palpation			22	16	10	12	16	0
Impaired movement of the mandible			21	23	11	10	9	4
Referral of pain within the head			22	12	6	9	6	5
Clicking in temporomandibular joint		Unilateral	32	4	9	10	6	4
		Bilateral	11	8	2	3	2	1

**Table 3:** The results of statistical analysis concerning reported values of VNRS.

Group	Examination		
	I	II	II
Study group			
$\bar{x}$ ± *s*	**5.589** ± 1.304	**2.054** ± 1.095	**0.410** ± 0.626
Min-max	3–8	0–4	0–2
Median	5	2	0
Control group			
$\bar{x}$ ± *s*	**5.436** ± 1.548	**2.571** ± 1.188	**1.303** ± 1.007
Min-max	2–9	0–5	0–4
Median	5	3	1
Mann–Whitney *U* test, *p* ≤ **0.005**	*p*=**0.5015**	*p*=**0.00048**	*p*=**0.00040**

## Abbreviations

ARS: Anterior repositioning splint
CG: Control group
DC/TMD: Diagnostic Criteria for Temporomandibular Disorders
DDwR: Disc displacement with reduction
SG: Study group
TMJ: Temporomandibular joint
VNRS: Verbal Numerical Rating Scale.
